# Patient Experiences With Outpatient Pharmacy Services in Hospitals Using Automated Pharmacy Systems: Cross-Sectional Study

**DOI:** 10.2196/80963

**Published:** 2026-03-06

**Authors:** Ali Jasem Buabbas, Fatemah Mohammad Alsaleh, Mariam Alsanafi, Maryam Ali Safar, Ahmad T Al-Sultan, Talal Ahmad Zaman

**Affiliations:** 1 Department of Community Medicine and Behavorial Sciences, College of Medicine, Kuwait University, Hawalli, Jabriya, 13009, Kuwait, +965 90968464; 2 Department of Pharmacy Practice, College of Pharmacy, Kuwait University, Jabriya, Kuwait; 3 Ministry of Health, Kuwait, Kuwait; 4 College of Medicine, University of Alexandria, Alexandria, Egypt

**Keywords:** automated pharmacy, pharmacy practice, patient experience, patient satisfaction, e-dispensing, e-prescribing

## Abstract

**Background:**

Advancements in health technology and the adoption of electronic systems in hospital pharmacies have transformed pharmacy practice and service delivery, with patients and health care providers reporting perceived benefits related to patient care and safety. Therefore, it is of paramount importance to seek patients’ opinions based on their experiences in receiving outpatient pharmacy services through automated pharmacy systems.

**Objective:**

This study aimed to investigate patients’ experiences and explore their satisfaction with outpatient pharmacy services in hospitals using automated pharmacy systems.

**Methods:**

This cross-sectional study used an online questionnaire to assess patients’ experiences of outpatient pharmacy services in governmental hospitals in Kuwait where automated pharmacy systems are implemented.

**Results:**

Almost 95% (393/416) of the participants either agreed or strongly agreed that they were totally satisfied with the services delivered through the automated pharmacy systems. Participants reported the perceived benefits of these outpatient pharmacy services, including perceived prevention of medication errors (211/416, 50.7%), time savings (356/416, 85.6%), and easier access to prescription information at any time (249/416, 59.9%).

**Conclusions:**

Patients’ experiences with outpatient pharmacy services in automated pharmacy settings were generally positive, reflecting satisfaction with medication collection and service quality.

## Introduction

Over the past years, pharmacy practice has undergone significant transformation with the adoption of electronic prescribing (e-prescribing) and electronic dispensing (e-dispensing; hospital information system [HIS]) systems, resulting in numerous advantages within professional practice settings. Notable examples include reductions in medication errors [1-4], time savings [5,6], and increased work efficiency [1,2,6]. However, such systems also present certain challenges. Reported drawbacks include risks to medication safety (eg, unclear or incorrect dosage instructions or strength) [4], technical issues [1,5], and resistance to adoption among staff [1]. Difficult adoption of automated systems has been reported in previous studies and has been attributed to their incapability to execute some functions that were previously completed manually [2,5,6]. The implementation of e-prescribing and/or e-dispensing systems has been extensively examined in the literature, with a focus on evaluating whether their intended purposes have been achieved and whether the anticipated benefits have been realized.

A systematic review of the literature on the barriers and facilitators to implementing an e-prescribing system highlighted the importance of identifying the perceptions of user groups, including pharmacists, managers, and patients [7]. Patients’ involvement in the assessment process is essential because the quality of outpatient pharmacy service delivery is ultimately perceived by them; therefore, patient satisfaction can be considered an indicator of success [8,9]. Previous studies have supported the need for future research to evaluate the impact of integrated e-prescribing and e-dispensing systems [8,10], as the combination of these 2 domains has the potential to improve various aspects of pharmacy schemes and practice [10].

In this study, the term “automated pharmacy” encompasses 2 key domains. The first is the receipt of prescriptions via an e-prescribing system, also referred to as a “pharmacy information system,” which is a subset of a much greater HIS. Within such a system, patient data and medication lists are stored electronically. The second component of the “automated pharmacy” concept involves an e-dispensing system, in which electronic cabinets with the drugs on the shelves (most of which are prepackaged) are used to deliver the prescribed medications to the dispensing area. Briefly, the pharmacist receives the prescription from the patient, reviews it to ensure that the medications are accurately prescribed, and uses the e-prescribing system to send the order to the electronic or automated cabinet. The cabinet prepares the medications and delivers them to the pharmacist, who then dispenses them and provides patient counseling.

In Kuwait, there are 6 general governmental hospitals, all of which have transitioned their outpatient pharmacies from manual processes to automated systems. However, the extent of implementation varies across institutions. In some settings, automated dispensing systems are not fully connected to the e-prescribing platform, while in others, the e-prescribing system functions as a stand-alone tool that is not integrated with the HIS or electronic health records.

Following implementation, several challenges arise for staff operating integrated e-prescribing and automated dispensing systems, despite the technological advancements they offer. These challenges include technical difficulties, workflow and organizational issues, and factors related to users’ attitudes, adaptation, and readiness to use technology. Such issues can affect the quality of the outpatient pharmacy services delivered to patients, particularly regarding waiting times, the adequacy of medication counseling, availability of medications, and the accuracy of medication dispensing. These challenges highlight the need to explore patients’ experiences to understand their perceptions and satisfaction with outpatient pharmacy care in hospitals, which can help identify areas for improving and optimizing service quality.

Within this context, previous studies in Kuwait have examined the perspectives of health care providers regarding the use of HISs in primary care settings [11,12] or secondary care settings [13]. Patients’ satisfaction with e-prescribing and electronic health records has also been explored [14,15]. In the existing literature, there is no research in Kuwait that examined patients’ satisfaction based on their experiences with integrated automated outpatient pharmacy services. Thus, this study aimed to assess patients’ satisfaction with their experiences in receiving outpatient pharmacy services through integrated e-prescribing and e-dispensing systems in the outpatient pharmacies of secondary care governmental hospitals. This enabled the development of recommendations to improve pharmacy practice in Kuwait.

## Methods

### Study Design, Sample, and Setting

The design of this study was a cross-sectional survey, using a questionnaire developed in Google Forms that was electronically administered to collect data from patients at their convenience between June and July 2024 at the outpatient pharmacies of 4 governmental hospitals in the state of Kuwait. The selected hospitals were Adan, Jahra, Farwaniya, and Jaber Al-Ahmad, which had all adopted and implemented the concept of automated pharmacy, which is using an integrated e-prescribing and e-dispensing system in the outpatient pharmacies.

### A Survey Questionnaire

The items of the questionnaire were taken from previous validated studies [16]. Content validity was evaluated by the research team, which included 2 academic staff members from the Pharmacy Practice Department at the College of Pharmacy, Kuwait University, who are experts in their field, and 1 academic staff member from the College of Medicine with expertise in health informatics. This evaluation involved reviewing the questionnaire items to ensure their relevance to the study objectives and their clarity. Several modifications were made to ensure compatibility and appropriateness for the research aim, sample, and setting. The term “medication teaching” was replaced with “medication counseling,” as this terminology is commonly used and understood by patients and pharmacy staff in Kuwait. The section “pharmacy place” was removed because it addressed the physical layout of the pharmacy, which was unrelated to the study’s aim and objectives. Additionally, 1 item was excluded, which was about “the availability of a private area for counseling,” because this service is not consistently provided across outpatient pharmacies in Kuwait and is not applicable to the local setting. A new section was added, which was about “dispensing medications,” and was developed from previous studies [17,18] to explore aspects of communication between pharmacists and patients.

### Questionnaire Structure

The questionnaire consisted of 40 items and seven sections: (1) time of prescription dispensing (3 items), (2) pharmacist attitudes (5 items), (3) medication supply (5 items), (4) medication counseling (5 items), (5) dispensing medications (5 items), (6) overall impact of automated pharmacy on outpatient pharmacy services (7 items), and (7) sociodemographic characteristics (10 items).

### Inclusion and Exclusion Criteria

This study included secondary care hospitals that offer integrated automated outpatient pharmacy services. Patients and/or caregivers who provide close, hands-on care to the patient are familiar with the patient’s experiences with outpatient pharmacy services and can offer valuable insights alongside the patients themselves. Eligible participants were those who were able to complete the questionnaire and were therefore invited to take part in the study. Private hospitals and private pharmacies were excluded from this study because they operate under different policies and procedures and do not use integrated e-prescribing and e-dispensing systems within their outpatient pharmacy services.

### Pilot Study

A pilot study was conducted to ensure clarity and readability with 25 patients who were waiting to receive their medications from outpatient pharmacies. The results demonstrated that the questionnaire was easy to understand and well received, confirming its readiness for full-scale distribution.

### Recruitment and Data Collection

**Figure 1.**
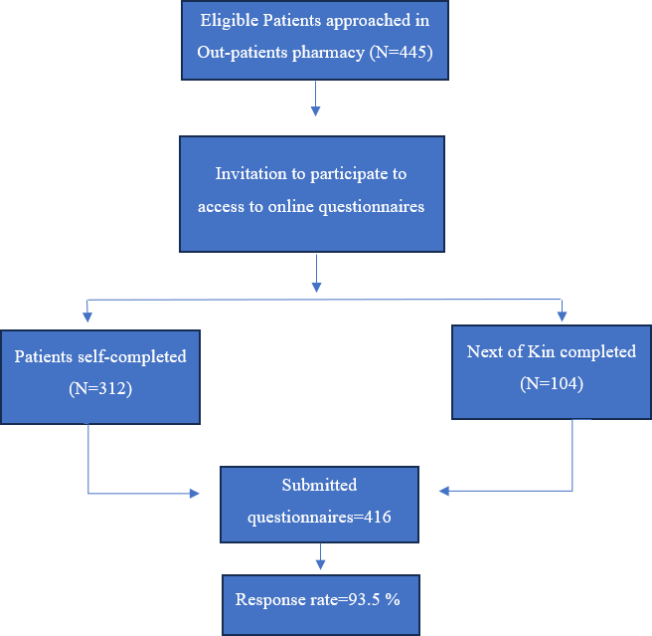
The recruitment protocol of the study participants.

Patients waiting to receive their medications in the outpatient pharmacy areas were approached and invited to participate voluntarily. The questionnaire was administered to patients using a QR code or via a tablet device, providing direct access to the online questionnaire. No identifying patient information was collected, and responses could not be linked to individual patients. Pharmacy staff were not present while participants completed the survey, which helped maintain anonymity and reduce potential social desirability bias. For patients unable to complete the questionnaire themselves, a next of kin who was familiar with their experience (eg, a spouse, son, or sister) was permitted to respond on their behalf (Figure 1).

The questionnaire was offered in 2 languages, Arabic and English, to accommodate a diverse patient population. The original English version of the questionnaire was translated into Arabic, and the translation was validated using a back-translation method to ensure accuracy and clarity. All patients who met the inclusion criteria were invited to participate. Data collection was undertaken by a group of pharmacy students (n=6) from the College of Pharmacy, Kuwait University. They were available in the waiting areas of the outpatient pharmacies to share the online questionnaire with patients.

### Construct Validation Using Factor Analysis and Reliability Assessment

The Kaiser-Meyer-Olkin (KMO) test and Bartlett’s test of sphericity were used to evaluate sampling adequacy [19]. These tests help determine whether data are suitable for factor analysis. Data factorability was confirmed with a KMO value above 0.50 and a significant Bartlett’s test value (*P*<.05) [19]. Principal component analysis with Promax rotation, an oblique method, was used. Promax was selected because it permits factors to correlate, which aligns with the expected relationships among the constructs [20]. The number of factors was decided based on eigenvalues, scree plots, and explained variance. Exploratory factor analysis was performed with a factor loading cutoff of 0.40 to extract scale dimensions [19,21].

Internal consistency reliability was assessed using Cronbach α coefficient based on a 4-point Likert scale (time of prescription dispensing, pharmacist attitudes, medication supply, medication counseling, and dispensing medications). A Cronbach α value of ≥0.70 was regarded as satisfactory evidence of internal consistency for the new instrument [19,20,22].

### Ethical Considerations

Ethical approval for this study was obtained from the The Standing Committee for Coordinating Medical and Health Research prior to data collection (reference number 2352). Participation was voluntary, and informed consent was obtained from all participants before administering the survey. To protect privacy and confidentiality, no personally identifiable information was collected. Surveys were completed anonymously in the outpatient pharmacy waiting area, and participants were informed that they could decline to answer any question or withdraw at any time without any impact on their care or pharmacy services. All collected data were kept confidential, stored securely, and accessible only to the research team. Study findings are reported in aggregate form, ensuring that no individual participant can be identified.

### Statistical Data Analysis

Statistical analyses were conducted using IBM SPSS Statistics (version 29; IBM Corp). Descriptive statistics were used to present the data. Categorical variables were presented as frequencies and percentages. To summarize the patients’ perspective scores, responses for the questionnaire items were averaged across all items within the category. As an initial exploratory comparison, patient and caregiver perspective scores were compared using the Mann-Whitney *U* test due to nonnormal score distributions and unequal group sizes.

A normality test for the component scores was undertaken using the Shapiro-Wilk test, which indicated that the data were not normally distributed. Accordingly, results are presented as minimum, maximum, median, and IQR. Nonparametric statistical tests were used to assess associations between component scores and variables of interest, including the Mann-Whitney *U* test for binary comparisons, the Kruskal-Wallis test for comparisons across multiple groups, Spearman rank correlation for continuous variables, and the chi-square test for trend for ordinal categorical variables.

For analyses based on a priori hypotheses involving comparisons across multiple groups, post hoc pairwise comparisons were conducted following statistically significant Kruskal-Wallis tests, with *P* values adjusted for multiple comparisons using the Dunn-Bonferroni method. These analyses were planned and considered confirmatory. Effect sizes were reported as rank-biserial correlation coefficients (r_rb) for binary comparisons, epsilon squared (ε²) for multigroup comparisons, and Spearman correlation coefficient (ρ) for continuous variables.

Multiple linear regression analyses were performed to identify independent factors associated with the overall score across domains of patients’ perspectives on automated pharmacy services. Additional association analyses not specified a priori were treated as exploratory and were not adjusted for multiple comparisons; these results are presented for hypothesis generation. Ordinal survey responses were averaged across items within each component to generate composite scores for inferential analyses. Statistical significance was set at *P* value of <.05.

A sensitivity analysis was conducted to assess the results to potential selection bias arising from voluntary participation and convenience sampling. This analysis examined the possible impact of underrepresentation of less satisfied patients on the study outcomes. Specifically, the patients’ perspective score was systematically reduced by 0.3, 0.5, and 1.0 SDs, representing mild, moderate, and severe dissatisfaction scenarios, respectively. These thresholds were selected based on established effect size conventions for continuous outcomes and their application in patient-reported outcome research [23]. For each scenario, the multiple linear regression analyses were repeated using the adjusted outcome variable, while all predictor variables and model specifications remained unchanged.

## Results

### Sociodemographic Characteristics of the Study Participants

A total of 445 patients were approached, of whom 416 completed the questionnaire, producing a response rate of 93.5%. The sociodemographic characteristics of the study participants are illustrated in Table 1.

**Table 1.** Sociodemographic characteristics of participants (N=416).

Characteristics	Values, n (%)
Total	416 (100.0)
Hospital	
Adan	111 (26.7)
Jahra	95 (22.8)
Farwaniya	100 (24.0)
Jaber Al-Ahmad	110 (26.4)
Age (years)	
<29	142 (34.3)
30-39	108 (26.1)
40-49	105 (25.4)
≥50	59 (14.3)
Nationality	
Kuwaiti	351 (84.4)
Non-Kuwaiti	65 (15.6)
Sex	
Male	117 (28.1)
Female	299 (71.9)
Marital status	
Single	158 (38.0)
Married	258 (62.0)
Education	
High school or less	76 (18.3)
Diploma	108 (26.0)
Bachelor	208 (50.0)
Higher education	24 (5.8)
Estimated number of pharmacy visits in the last year	
Once to twice	92 (22.1)
3-4 times	155 (37.3)
Monthly	161 (38.7)
Weekly	8 (1.9)
Dispense medications	
Personally	312 (75.0)
Someone else	104 (25.0)
The time I have waited to receive my medication	
≤5 minutes	136 (35.5)
6-15 minutes	167 (43.6)
>15 minutes	80 (20.9)
The number of medications in my prescription	
1-5 items	331 (79.6)
6-10 items	62 (14.9)
>10 items	23 (5.5)

Most participants were female (299/416, 71.9%), Kuwaiti nationals (351/416, 84.4%), younger than 29 years (142/416, 34.3%), and married (258/416, 62.0%). Half of the participants (208/416, 50%) held a bachelor’s degree. Regarding pharmacy visits, approximately one-third of the participants visited the pharmacy monthly (161/416, 38.7%), while another third reported visiting 3 or 4 times per year (155/416, 37.3%). Most participants were collecting medications for themselves (312/416, 75%) and had between 1 and 5 medications in their prescriptions (331/416, 79.6%). Dispensing waiting times varied, with the majority of participants waiting 6‐15 minutes (167/416, 40.1%), followed by those waiting less than 5 minutes (136/416, 32.7%), and those waiting more than 15 minutes (80/416, 19.2%).

Based on the exploratory factor analysis results shown in Table 2, each item with a value greater than 0.40 was assigned to an extracted factor. The KMO value of 0.914 indicated that the data were appropriate for factor analysis, and the value of Bartlett’s test of sphericity was significant (*χ*^2^_231_=5140.38; *P*<.001). The scree plot indicated a 5-component solution, with all items showing acceptable communalities, and eigenvalues greater than 1.0 were extracted, accounting for 67.27% of the total variance. The reliability of the 22-item factored scale was obtained. The overall Cronbach α for the scale showed excellent reliability (α=0.92).

**Table 2.** Results of the exploratory factor analysis.

Component	Item	Factor loadings^a^	Eigenvalue	VE^b^ (%)	CVE^c^ (%)
Time of prescription dispensing	1.79	8.12	8.12
	1.00	0.83			
2.00	0.90
Pharmacist attitude	4.03	18.32	26.44
	3.00	0.74			
4.00	0.74
5.00	0.83
6.00	0.80
7.00	0.75
Medication supply	3.56	16.18	42.62
	8.00	0.67			
9.00	0.62
10.00	0.72
11.00	0.73
12.00	0.71
Medication counseling	3.05	13.84	56.46
	13.00	0.52			
14.00	0.40
15.00	0.84
16.00	0.85
17.00	0.68
Dispensing medication	2.38	10.81	67.27
	18.00	0.43			
19.00	0.79
20.00	0.69
21.00	0.79
22.00	0.54

a Extraction method: principal component analysis. Rotation method: Promax. Factor loading cutoff: >0.40.

b VE: variance explained.

c CVE: cumulative variance explained.

### Time of Prescription Dispensing

The results showed that most respondents agreed or strongly agreed that their prescriptions were dispensed within a reasonable time (364/416, 87.5%; Table 3).

**Table 3.** Perceptions of e-prescription and automated dispensing systems in outpatient pharmacies at secondary care hospitals (N=416).

Item	Values, n (%)								Range	Overall median score (IQR)^a^
	Strongly agree	Agree	Disagree	Strongly disagree	Never	Rarely	Sometimes	Many times		
Time of prescription dispensing									1.0-4.0	3.0 (0.5)
1. Receive medications within a reasonable time	136 (32.7)	228 (54.8)	38 (9.1)	14 (3.4)	—^b^	—	—	—		
2. Waiting time is acceptable considering the quantity of prescription medication	115 (27.6)	226 (54.3)	63 (15.1)	12 (2.9)	—	—	—	—		
Pharmacist attitude									4.0-1.2	3.4 (1.0)
3. Pharmacist helped me to get the medications	201 (48.3)	201 (48.3)	9 (2.2)	5 (1.2)	—	—	—	—		
4. Pharmacist helped to solve any problem while getting the medication	168 (40.4)	211 (50.7)	33 (7.9)	4 (1.0)						
5. Pharmacist answered my questions	197 (47.4)	200 (48.1)	18 (4.3)	1 (0.2)						
6. Pharmacist understood the medical case	172 (41.3)	222 (53.4)	21 (5.0)	1 (0.2)						
7. Pharmacist treated me with respect	254 (61.1)	153 (36.8)	5 (1.2)	4 (1.0)						
Medication supply									4.0-1.8	3.2 (0.8)
8. Medication quantity was sufficient	178 (42.8)	214 (51.4)	19 (4.6)	5 (1.2)						
9. All my medications were available in the pharmacy	131 (31.5)	180 (43.3)	87 (20.9)	18 (4.3)						
10. Medication name was clear and easy to read	197 (47.4)	200 (48.1)	14 (3.4)	5 (1.2)						
11. Medication label or sticker instructions were clear	192 (46.2)	202 (48.6)	18 (4.3)	4 (1.0)						
12. Medication appearance and quality was good	195 (46.9)	211 (50.7)	10 (2.4)	0 (0.0)						
Medication counseling									4.0-1.0	3.0 (0.8)
13. Pharmacist explained the reason of my medication	169 (40.6)	187 (45.0)	44 (10.6)	16 (3.8)						
14. Pharmacist told me how to take the correct medication dose	189 (45.4)	192 (46.2)	27 (6.5)	8 (1.9)						
15. Pharmacist explained my medication’s possible side effects	84 (12.5)	159 (38.2)	159 (38.2)	52 (12.5)						
16. Pharmacist explained how to store my medication	82 (12.5)	128 (30.8)	154 (37.0)	52 (12.5)						
17. I had enough time with the pharmacist	123 (29.6)	194 (46.6)	77 (18.5)	22 (5.3)						
Dispensing medication									4.0-1.0	3.8 (0.4)
18. The communication with the pharmacist and/or the doctor was challenging	—	—	—	—	218 (52.4)	111 (26.7)	71 (17.1)	16 (3.8)		
19. The prescription was sent to the wrong pharmacy	—	—	—	—	349 (83.9)	53 (12.7)	12 (2.9)	2 (0.5)		
20. The wrong medication was dispensed to me	—	—	—	—	384 (92.3)	26 (6.3)	4 (1.0)	2 (0.5)		
21. I received wrong instructions regarding the drugs dispensed for me	—	—	—	—	371 (89.2)	31 (7.5)	12 (2.9)	2 (0.5)		
22. My medical prescription or information was not received by the pharmacy	—	—	—	—	294 (70.7)	79 (19.0)	32 (7.7)	11 (2.6)		

a For each perspective score, responses for the questionnaire items were averaged across all items within the category.

b Not applicable.

Most participants (341/416, 82.0%) also found the waiting time acceptable, considering the number of medications prescribed. A statistically significant association (*P*=.006; chi-square test) was found between the number of medications prescribed and the reported waiting time (Table 4). Patients prescribed 1‐5 medications were more likely to experience shorter waiting times: 38.8% (118/304) received their medications within 5 minutes, 41.8% (127/304) waited 6‐15 minutes, and 19.4% (59/304) waited more than 15 minutes.

**Table 4.** Association between the number of medications and the waiting time to receive the medications (*P*=.006^a^; chi-square for trend).

Number of medications	Waiting time		
≤5 minutes	6-15 minutes	>15 minutes
1-5 items, n (raw %)	118 (38.8)	127 (41.8)	59 (19.4)
6-10 items, n (raw %)	14 (24)	31 (53)	13 (22)
>10 items, n (raw %)	4 (19)	9 (43)	8 (38)

a Chi-square test for trend.

In contrast, patients prescribed 6‐10 medications experienced longer waiting times. Only 24% (14/58) received their medications within 5 minutes, while 53% (31/58) waited 6‐15 minutes, and 22% (13/58) waited more than 15 minutes. Among those prescribed more than 10 medications, few (4/21, 19%) received their medications within 5 minutes or less, while many (9/21, 43%) waited 6‐15 minutes, and a similar proportion (8/21, 38%) waited more than 15 minutes, as shown in Table 4.

### Pharmacist Attitudes

As shown in Table 3, the results indicated that most participants had positive perceptions of pharmacist attitudes. The majority agreed or strongly agreed that the pharmacists helped obtain their medications (401/416, 96.5%) and assisted them in resolving issues related to medication access (379/416, 91.1%). In addition, a large proportion reported that the pharmacists answered their questions (397/416, 95.5%) and demonstrated knowledge and understanding toward their medical cases (394/416, 94.7%). Furthermore, most participants shared the perspective that the pharmacists treated them with respect and dignity (407/416, 97.9%).

### Medication Supply

Most participants found the quantities of medications dispensed to be sufficient (392/416, 94.2%). Additionally, nearly three-quarters (311/416, 74.8%) reported that all their prescribed medications were available in the pharmacy (Table 3). However, a subset of participants (105/416, 25.2%) encountered instances where 1 or more prescribed medications were unavailable. Medication labels were clear and easy to read for most participants (397/416, 95.5%), and the usage instructions were clear and understandable (394/416, 94.8%). Furthermore, the majority expressed satisfaction with the appearance and quality of the medications provided (406/416, 97.6%).

### Medication Counseling

The results revealed that the pharmacists performed well in several key areas of patient counseling (Table 3), which included explaining the purpose of medication use (356/416, 85.6%), providing instructions on correct medication dosage (381/416, 91.6%), and dedicating sufficient time to patients during consultations (317/416, 76.2%). However, notable gaps were identified in some specific aspects of counseling. Only half of the participants reported receiving information about the potential side effects of their medications (211/416, 50.7%). Similarly, counseling on proper medication storage was lacking, with approximately half of the participants not receiving such explanations (206/416, 49.5%).

### Dispensing Medications

As shown in Table 3, most participants reported that communication challenges with pharmacists and/or physicians rarely or never occurred (329/416, 79.1%). Similarly, errors in the e-prescribing and e-dispensing process were uncommon. Specifically, the majority of participants agreed that rarely or never were prescriptions sent to the wrong pharmacy (402/416, 96.6%), the wrong medication was dispensed (410/416, 98.6%), incorrect instructions were received regarding the medications dispensed (402/416, 96.7%), or the prescription information was not received by the pharmacy (373/416, 89.7%).

### Impact of Automated Pharmacy and Overall Satisfaction With Outpatient Pharmacy Services

The participants identified several perceived benefits of the integrated e-prescribing and e-dispensing systems, reflecting their positive impact on outpatient pharmacy services (Multimedia Appendix 1). These perceived benefits included time savings (356/416, 85.6%), perceived prevention of medication errors (211/416, 50.7%), and convenient access to prescription information at any time (249/416, 59.9%).

Also, the results showed high overall satisfaction with the automated outpatient pharmacy services, as 94.5% (393/416) of participants either agreed or strongly agreed that they were satisfied with the services delivered through the automated systems (Multimedia Appendix 2).

The overall median score for the time of prescription dispensing section was 3.0, while the median score for pharmacist attitudes was 3.4. The median scores for the medication supply, medication counseling, and dispensing medications domains were 3.2, 3.0, and 3.8, respectively (Table 3).

Tables5  6 demonstrate the relationships between sociodemographic characteristics and the overall median scores for each questionnaire component. There were significant differences among hospitals, with small effect sizes for the time of prescription dispensing and the dispensing medications scores (ε^2^=0.05 and ε^2^=0.02, respectively). The median time of prescription dispensing score for Farwaniya hospital was significantly higher than for Adan hospital (3.2 vs 3.0; adjusted *P*=.03) and Jaber Al-Ahmad hospital (3.2 vs 3.0; adjusted *P*<.001). The median dispensing medications score for Jaber Al-Ahmad hospital was significantly higher than for Adan hospital (4.0 vs 3.8; adjusted *P*=.02) and Jahra hospital (4.0 vs 3.8; adjusted *P*=.04).

**Table 5.** Component scores for a survey assessing e-prescribing and e-dispensing systems in outpatient pharmacies at secondary care hospitals according to sociodemographic characteristics (N=416).

Characteristics	Median score (IQR)^a^				
	Time of prescription dispensing	Pharmacist attitude	Medication supply	Medication counseling	Dispensing medication
Hospital					
Adan	3.0 (0.5)	3.4 (1.0)	3.2 (0.8)	3.0 (0.8)	3.8 (0.6)
Jahra	3.0 (1.0)	3.4 (1.0)	3.2 (1.0)	3.0 (1.0)	3.8 (0.6)
Farwaniya	3.2 (1.0)	3.4 (1.0)	3.3 (1.0)	3.0 (1.2)	3.8 (0.4)
Jaber Al-Ahmad	3.0 (1.5)	3.6 (1.0)	3.4 (0.8)	3.0 (0.9)	4.0 (0.4)
Age (years)					
<29	3.0 (0.6)	3.4 (1.0)	3.2 (1.0)	3.0 (1.0)	3.8 (0.6)
30-39	3.0 (1.0)	3.6 (1.0)	3.3 (0.8)	3.0 (0.9)	3.8 (0.4)
40-49	3.0 (0.5)	3.4 (1.0)	3.2 (0.8)	3.0 (0.8)	3.8 (0.4)
≥50	3.0 (0.5)	3.4 (0.8)	3.2 (0.8)	2.6 (1.0)	4.0 (0.4)
Nationality					
Kuwaiti	3.0 (0.5)	3.4 (1.0)	3.2 (0.8)	3.0 (1.0)	3.8 (0.6)
Non-Kuwaiti	3.0 (0.8)	3.6 (1.0)	3.2 (0.9)	3.0 (1.1)	3.8 (0.4)
Sex					
Male	3.0 (1.0)	3.6 (1.0)	3.4 (1.0)	3.0 (1.2)	3.8 (0.6)
Female	3.0 (0.5)	3.4 (1.0)	3.2 (0.8)	3.0 (0.8)	3.8 (0.4)
Marital status					
Single	3.0 (0.5)	3.5 (1.0)	3.2 (0.8)	3.0 (0.8)	3.8 (0.6)
Married	3.0 (0.5)	3.4 (1.0)	3.2 (0.8)	3.0 (1.0)	3.8 (0.4)
Education					
High school or less	3.0 (1.0)	3.6 (1.0)	3.2 (1.0)	3.0 (1.0)	4.0 (0.4)
Diploma	3.0 (0.5)	3.2 (1.0)	3.4 (0.8)	3.0 (1.4)	3.8 (0.6)
Bachelor	3.0 (0.5)	3.4 (1.0)	3.2 (0.8)	3.0 (0.8)	3.8 (0.6)
Higher education	3.0 (1.9)	3.7 (0.9)	3.6 (1.0)	3.1 (1.6)	3.9 (0.9)
Estimated number of pharmacy visits in the last year					
Once to twice	3.0 (1.4)	3.4 (1.0)	3.1 (1.0)	3.0 (1.0)	4.0 (0.6)
3-4 times	3.0 (0.5)	3.4 (1.0)	3.4 (0.8)	3.0 (0.8)	3.8 (0.4)
Monthly	3.0 (0.5)	3.6 (1.0)	3.2 (0.8)	3.0 (1.0)	3.8 (0.6)
Weekly	3.0 (1.0)	3.3 (1.4)	3.0 (1.1)	2.5 (1.8)	3.6 (0.9)
Dispense medications					
Personally	3.0 (0.5)	3.4 (1.0)	3.2 (0.8)	3.0 (0.8)	3.8 (0.4)
Someone else	3.0 (0.5)	3.3 (1.0)	3.2 (0.9)	2.9 (1.0)	3.8 (0.8)
The time I have waited to receive my medication					
≤5 minutes	3.5 (1.0)	3.6 (0.9)	3.6 (1.0)	3.0 (1.0)	4.0 (0.4)
6-15 minutes	3.0 (0.5)	3.4 (0.8)	3.2 (0.6)	2.8 (0.6)	3.8 (0.6)
>15 minutes	2.8 (1.0)	3.2 (1.0)	3.1 (1.0)	2.8 (1.0)	3.6 (0.6)
The number of medications in my prescription					
1-5 items	3.0 (0.5)	3.4 (1.0)	3.4 (0.8)	3.0 (0.8)	3.8 (0.4)
6-10 items	3.0 (0.5)	3.5 (1.0)	3.0 (1.0)	3.0 (0.9)	3.8 (0.6)
11-15 items	3.0 (1.0)	3.0 (0.6)	3.2 (0.8)	3.0 (1.0)	3.8 (0.6)

a For each perspective score, responses for the questionnaire items were averaged across all items within the category.

**Table 6.** Relationships between sociodemographic variables and component scores for a survey assessing e-prescribing and e-dispensing systems in outpatient pharmacies at secondary care hospitals (N=416).

Characteristics	Time of prescription dispensing		Pharmacist attitude		Medication supply		Medication counseling		Dispensing medication	
Effect size^a^	*P* value^b^	Effect size^a^	*P* value^b^	Effect size^a^	*P* value^b^	Effect size^a^	*P* value^b^	Effect size^a^	*P* value^b^
Hospital	0.055	<.001	0.004	.71	0.004	.73	0.003	.60	0.018	.02
Age (years)	−0.050	.31	−0.050	.34	−0.003	.96	−0.130	.007	0.040	.46
Nationality	0.370	.72	−0.120	.90	0.150	.89	1.370	.17	0.450	.65
Gender	0.219	.13	0.225	.12	0.105	.09	0.053	.39	0.112	.06
Marital status	0.028	.61	0.008	.89	0.019	.74	0.057	.33	0.052	.35
Education	0.110	.03	0.010	.87	0.040	.43	−0.070	.15	0.090	.07
Number of visits (last year)	−0.010	.89	0.030	.54	0.001	.98	0.030	.63	−0.060	.22
Dispense medications	0.281	.12	0.242	.46	0.266	.22	0.272	.190	0.299	.06
Time waited	−0.460	<.001	−0.150	.003	−0.150	.003	−0.220	<.001	−0.170	.001
Number of medications	−0.110	.03	−0.030	.51	−0.090	.06	−0.020	.77	−0.050	.33

a Rank-biserial correlation (r_rb_) for 2-group comparisons, epsilon squared (ε2) for multigroup comparisons, and Spearman correlation coefficient for continuous variables. Interpretation thresholds: *r*_rb_≈0.10=small, ≈0.30=moderate, and ≥0.50=large; ε2≈0.01=small, ≈0.06=moderate, and ≥0.14=large; and value of *Ρ* ranges from −1 to +1, *Ρ*<.1=small, .1-.3=medium, and >.5=large.

b Mann-Whitney *U* tests (2 groups), Kruskal-Wallis tests (≥3 groups), or Spearman rank correlation test (continuous variables).

An indirect correlation coefficient of *r*=−0.13 was present between age and medication counseling score (*P*=.007). Likewise, there was an indirect correlation between dispensing medications score and education level (*r*=−0.11; *P*=.03). All 4 other domains of the questionnaire had scores that were significantly related to the amount of time it took to receive prescriptions (*P*<.05), with indirect Spearman rank correlation coefficients ranging from −0.15 to −0.46. Furthermore, a significant indirect correlation coefficient of *r*=−0.11 was found between number of medications and dispensing medications score (*P*=.03). For other patient characteristics, no statistically significant differences or associations were observed across any of the 5 perspective domains (all *P*>.05).

All 5 domains of the questionnaire were significantly related to each other (*P*≤.001), with Spearman rank correlation coefficients ranging from 0.30 to 0.62 (Table 7). The number of medications a patient was prescribed and the amount of time they waited to obtain those prescriptions had a significant association (*P*=.006), as seen in Table 4. In other words, the amount of waiting time rises as the number of medications increases (from 1‐5 items to 6‐10 items or 10 items or more).

**Table 7.** Spearman rank correlations between domains in a survey assessing e-prescribing and e-dispensing systems in outpatient pharmacies at secondary care hospitals (N=416).

Component	Time of prescription dispensing		Pharmacist attitude		Medication supply		Medication counseling		Dispensing medication	
*R*	*P* value	*R*	*P* value	*R*	*P* value	*R*	*P* value	*R*	*P* value
Time of prescription dispensing	N/A^a^	N/A	0.48	<.001	0.47	<.001	0.44	<.001	0.3	<.001
Pharmacist attitude	0.48	<.001	N/A	N/A	0.62	<.001	0.62	<.001	0.39	<.001
Medication supply	0.47	<.001	0.62	<.001	N/A	N/A	0.62	<.001	0.48	<.001
Medication counseling	0.44	<.001	0.62	<.001	0.62	<.001	N/A	N/A	0.48	<.001
Dispensing medication	0.3	<.001	0.39	<.001	0.48	<.001	0.48	<.001	N/A	N/A

a Not applicable.

The results showed that there is an inverse relationship between medication dispensing waiting time and patient perceptions of automated outpatient pharmacy services, whereas increased waiting times were associated with less satisfaction of pharmacists’ attitudes, medication counseling, and dispensing practices.

Table 8 shows the results of the multiple regression model examining patients’ perceptions toward automated outpatient pharmacy services, with total perception scores modeled as a function of sociodemographic and other relevant variables. All variables were entered simultaneously to estimate their independent associations. The model was statistically significant (*F*_231_=2.32; *P*=.001) and explained 11% of the variance in patients’ perceptions. Compared with patients who waited 5 minutes or less, those who waited 6-15 minutes and more than 15 minutes reported significantly lower perception scores (*β*=−3.70, 95% CI −5.76 to −1.65, *P*<.001 and *β*=−6.19, 95% CI −8.82 to −3.56, *P*< .001, respectively).

**Table 8.** Multivariable linear regression predicting the total score across all key domains of patients' perspectives on automated pharmacy, adjusted for sociodemographic and other variables.

Characteristics	Regression coefficient (*β*)^a^	95% CI	SE	*t* statistic (*df*)	*P* value
Constant	78.69	74.42 to 82.96	2.17	36.22 (395)	<.001
Hospital					
Adan	N/A^b^	N/A	N/A	N/A	N/A
Jahraa	−1.57	−4.20 to 1.07	1.34	−1.17 (395)	.24
Farwaniya	−1.27	−3.95 to 1.41	1.36	−0.93 (395)	.35
Jaber AlAhmad	0.69	−1.88 to 3.25	1.30	0.53 (395)	.59
Age (years)					
<29	N/A	N/A	N/A	N/A	N/A
30‐39	−0.08	−2.61 to 2.46	1.29	−0.06 (395)	.95
40‐49	−1.40	−4.14 to 1.34	1.39	−1.01 (395)	.32
≥50	−1.83	−5.15 to 1.49	1.69	−1.08 (395)	.28
Nationality					
Kuwaiti	−0.95	−3.50 to 1.60	1.30	−0.73 (395)	.47
Non-Kuwaiti	N/A	N/A	N/A	N/A	N/A
Sex					
Male	N/A	N/A	N/A	N/A	N/A
Female	−0.68	−2.69 to 1.34	1.03	−0.66 (395)	.51
Marital status					
Single	N/A	N/A	N/A	N/A	N/A
Married	1.11	−1.14 to 3.36	1.15	0.97 (395)	.33
Education					
High school and less	N/A	N/A	N/A	N/A	N/A
Diploma	−1.23	−3.97 to 1.52	1.40	−0.88 (395)	.38
Bachelor	−2.16	−4.66 to 0.34	1.27	−1.70 (395)	.09
Higher education	−0.62	−4.93 to 3.69	2.19	−0.28 (395)	.78
Estimated number of pharmacy visits in the last year					
Once to twice	N/A	N/A	N/A	N/A	N/A
3-4 times	0.75	−1.64 to 3.13	1.21	0.62 (395)	.54
Monthly	1.74	−0.71 to 4.19	1.25	1.39 (395)	.16
Weekly	−2.96	−9.78 to 3.86	3.47	−0.85 (395)	.39
Dispense medications					
Personally	N/A	N/A	N/A	N/A	N/A
Someone else	−1.86	−3.94 to 0.21	1.06	−1.76 (395)	.08
The time I have waited to receive my medication					
≤5 minutes	N/A	N/A	N/A	N/A	N/A
6‐15 minutes	−3.70	−5.76 to −1.65	1.04	−3.55 (395)	<.001
>15 minutes	−6.19	−8.82 to −3.56	1.34	−4.63 (395)	<.001
The number of medications in my prescription					
1‐5 items	N/A	N/A	N/A	N/A	N/A
6‐10 items	−0.23	−2.81 to 2.34	1.31	−0.18 (395)	.86
>10 items	−2.58	−6.58 to 1.42	2.03	−1.27 (395)	.21

a Results from multivariable linear regression using the total score across all key components of patients' perspectives on automated pharmacy as a continuous outcome variable, along with hospital location, age, nationality, gender, marital status, education, number of pharmacy visits in the last year, dispensed medications, time waited to receive medication, and number of medications in the prescription. The regression coefficients (*β*) represent the mean difference in the overall patients' perspective score associated with each category increase, holding all other variables constant. The 95% CIs and *P* values are reported.

b Not applicable.

The results of the sensitivity analyses are shown in Table 9. Across all simulated dissatisfaction scenarios (−0.3 SD, −0.5 SD, and −1.0 SD), the direction, magnitude, and statistical significance of the regression coefficients remained consistent with those observed in the primary analysis (Table 8). In particular, the association between longer waiting times and lower patient perspective scores persisted across all scenarios, with nearly identical regression coefficients and 95% CIs. As expected, the model intercept decreased progressively with increasing downward shifts, reflecting lower assumed overall patient perspectives under more conservative assumptions. The results showed that there were no remarkable changes in the estimated effects of individual predictors, and no variables changed their significance status across the sensitivity analyses.

**Table 9.** Sensitivity analysis of multivariable linear regression outcomes across different dissatisfaction assumptions.

Characteristics	Mild (SD −0.3)			Moderate (SD −0.5)			Severe (SD −1.0)		
	*B*	95% CI	*P* value	*B*	95% CI	*P* value	*B*	95% CI	*P* value
Constant	75.9	71.6 to 80.1	<.001	74.0	69.7 to 78.3	<.001	69.3	65.0 to 73.6	<.001
Hospital									
Adan	N/A^a^	N/A	N/A	N/A	N/A	N/A	N/A	N/A	N/A
Jahraa	−1.6	−4.2 to 1.1	.24	−1.6	−4.2 to 1.1	.24	−1.6	−4.2 to 1.1	.24
Farwaniya	−1.3	−3.9 to 1.4	.35	−1.3	−3.9 to 1.4	.35	−1.3	−3.9 to 1.4	.35
Jaber AlAhmad	0.7	−1.9 to 3.3	.59	0.7	−1.9 to 3.3	.59	0.7	−1.9 to 3.3	.59
Age (years)									
<29	N/A	N/A	N/A	N/A	N/A	N/A	N/A	N/A	N/A
30‐39	−0.1	−2.6 to 2.5	.95	−0.1	−2.6 to 2.5	.95	−0.1	−2.6 to 2.5	.95
40‐49	−1.4	−4.1 to 1.3	.32	−1.4	−4.1 to 1.3	.32	−1.4	−4.1 to 1.3	.32
≥50	−1.8	−5.1 to 1.5	.28	−1.8	−5.1 to 1.5	.28	−1.8	−5.1 to 1.5	.28
Nationality									
Kuwaiti	−0.9	−3.5 to 1.6	.47	−0.9	−3.5 to 1.6	.47	−0.9	−3.5 to 1.6	.47
Non-Kuwaiti	N/A	N/A	N/A	N/A	N/A	N/A	N/A	N/A	N/A
Sex									
Male	N/A	N/A	N/A	N/A	N/A	N/A	N/A	N/A	N/A
Female	−0.7	−2.7 to 1.3	.51	−0.7	−2.7 to 1.3	.51	−0.7	−2.7 to 1.3	.51
Marital status									
Single	N/A	N/A	N/A	N/A	N/A	N/A	N/A	N/A	N/A
Married	1.1	−1.1 to 3.4	.33	1.1	−1.1 to 3.4	.33	1.1	−1.1 to 3.4	.33
Education									
High school and less	N/A	N/A	N/A	N/A	N/A	N/A	N/A	N/A	N/A
Diploma	−1.2	−4.0 to 1.5	.38	−1.2	−4.0 to 1.5	.38	−1.2	−4.0 to 1.5	.38
Bachelor	−2.2	−4.7 to 0.3	.09	−2.2	−4.7 to 0.3	.09	−2.2	−4.7 to 0.3	.09
Higher education	−0.6	−4.9 to 3.7	.78	−0.6	−4.9 to 3.7	.78	−0.6	−4.9 to 3.7	.78
Estimated number of pharmacy visits in the last year									
Once to twice	N/A	N/A	N/A	N/A	N/A	N/A	N/A	N/A	N/A
3-4 times	0.7	−1.6 to 3.1	.54	0.7	−1.6 to 3.1	.538	0.7	−1.6 to 3.1	.54
Monthly	1.7	−0.7 to 4.2	.16	1.7	−0.7 to 4.2	.164	1.7	−0.7 to 4.2	.16
Weekly	−3.0	−9.8 to 3.9	.39	−3.0	−9.8 to 3.9	.394	−3.0	−9.8 to 3.9	.39
Dispense medications									
Personally	N/A	N/A	N/A	N/A	N/A	N/A	N/A	N/A	N/A
Someone else	−1.9	−3.9 to 0.2	.08	−1.9	−3.9 to 0.2	.08	−1.9	−3.9 to 0.2	.08
The time I have waited to receive my medication									
≤5 minutes	N/A	N/A	N/A	N/A	N/A	N/A	N/A	N/A	N/A
6‐15 minutes	−3.7	−5.8 to −1.7	<.001	−3.7	−5.8 to −1.7	<.001	−3.7	−5.8 to −1.7]	<.001
>15 minutes	−6.2	−8.8 to −3.6	<.001	−6.2	−8.8 to −3.6	<.001	−6.2	−8.8 to −3.6]	<.001
The number of medications in my prescription									
1‐5 items	N/A	N/A	N/A	N/A	N/A	N/A	N/A	N/A	N/A
6‐10 items	−0.2	−2.8 to 2.3	.86	−0.2	−2.8 to 2.3	.86	−0.2	−2.8 to 2.3	.86
>10 items	−2.6	−6.6 to 1.4	.21	−2.6	−6.6 to 1.4	.21	−2.6	−6.6 to 1.4	.21

a Not applicable.

## Discussion

### Principal Findings

Studying patients’ opinions and satisfaction provides a clear picture of the perceived impact and success of implementing automated pharmacy systems in the real world. Patient feedback can help determine whether the system meets their needs, improves the quality of outpatient pharmacy services, and achieves its intended goals in practice.

The existing literature has shown numerous advantages of using such technology in pharmacy practice. In this context, this study revealed that patients perceived integrated e-prescribing and e-dispensing systems as beneficial to outpatient pharmacy services and expressed overall satisfaction with their experiences of care, as reported by 95% (393/416) of the participants. Participants cited several key perceived benefits, including time savings, perceived prevention of medication errors, convenient access to prescription information at any time, and effective communication between pharmacists and physicians. These findings are consistent with previous studies, which have similarly reported a perceived reduction in medication errors [1-3], time savings in the prescribing and dispensing process [5,6], and increased work efficiency [1,2,6] following the adoption of automated pharmacy systems.

### Influence of Sociodemographic Data on Participant Satisfaction

The findings revealed that the satisfaction levels of the participants varied across several sociodemographic groups. This study found an indirect association between age and medication counseling and between dispensing medications score and educational level. This could be explained by the notion that people with higher education levels could have higher expectations of health care services, which could lead to dissatisfaction when these are unmet [24]. Similar findings were found in a previous study [25], which assessed patient satisfaction toward outpatient pharmacy services among outpatients attending public health clinics in Malaysia. The findings revealed that demographic factors (eg, age, education level, self-perceived health status, and general knowledge of pharmacists) significantly influenced patients’ perceptions of health care services. Notably, older age and higher education levels were associated with a lower patient satisfaction mean score [25]. Similarly, a study conducted in Pakistan [26] reported that male patients were significantly more satisfied with community outpatient pharmacy services than females. Younger patients (aged 18‐27 years) exhibited greater satisfaction than older age groups. Illiterate and less-educated individuals showed greater satisfaction levels, while those with higher education levels were less satisfied, likely due to higher expectations. Marital status and occupation also played a potential role, with married individuals showing greater satisfaction and government employees showing less satisfaction. No significant association was observed among students after statistical adjustment [26].

### Patients’ Satisfaction With Dispensing Time, Pharmacists’ Attitudes, and Medication Supply

Although most patients in this study agreed that their prescriptions were dispensed in a reasonable time, the findings revealed that the patients with more medications experienced longer waiting times, suggesting challenges in processing large or complex orders using the automated pharmacy systems.

These findings aligned with those of a previous study into primary health care settings in Kuwait [27], which found that patients’ satisfaction was strongly influenced by the timeliness of prescription fulfillment by physicians. Numerous studies have demonstrated that e-prescription systems reduce dispensing times and improve pharmacy workflow [28]. In this context, a study assessed the impact of e-prescription systems on pharmacy workflow and practice in Finland and found a reduction in both median and average delivery times across various prescription types [29]. In particular, the maximum dispensing time with the adoption of e-prescribing systems was shorter in 2012 (6 minutes and 22 seconds) than the time with the use of non–e-prescribing systems in 2006 (12 minutes and 40 seconds). The study concluded that e-prescribing systems significantly improved the efficiency of prescription processing in Finnish community pharmacies [29]. Additionally, a study conducted in Sweden into the impact of integrated e-prescribing systems on pharmacists’ practice found that the pharmacists perceived the system as expediting prescription processing and reducing the risk of medication errors [1]. The results showed a positive correlation between the perceived usefulness of the electronic system for improving work efficiency and medication safety.

This study revealed that the participants were highly satisfied with the pharmacists’ attitudes. Nearly all the participants agreed that the pharmacists helped obtain their medications, effectively resolved issues related to medication access, answered their questions, demonstrated knowledge of their medical conditions, and treated them with dignity and respect. These findings were supported by previous studies showing that e-prescribing systems enhance communication between patients and pharmacists [7], patients and prescribers [5], and pharmacists and other health care providers [30,31]. Similarly, a study conducted in Qatar reported comparable levels of satisfaction among participants regarding pharmacists’ empathy and communication skills [16]. Furthermore, effective communication, regardless of whether systems are manual or electronic, has been found to significantly contribute to overall patient satisfaction [7,32].

The findings of this study demonstrated statistically significant differences in patient satisfaction across outpatient pharmacies in the 4 general hospitals, particularly regarding medication dispensing speed and pharmacists’ attitudes. These site-level variations may reflect differences in staffing levels, staff competencies, workload, maturity of automation, or local operational workflows. For instance, the higher satisfaction with medication dispensing speed observed at Farwaniya Hospital compared with Adan Hospital may be attributable to shorter waiting times, fewer medications per prescription, or more efficient dispensing practices. While patient satisfaction is an important indicator of service performance, it should be interpreted alongside technical and organizational factors to provide a comprehensive understanding of the effectiveness and efficiency of digital pharmacy systems.

The findings of this study indicated that approximately one-quarter of the surveyed participants experienced medication unavailability. Similar findings have been reported by previous studies [33-36], highlighting the need for improved inventory integration to overcome such issues and enhance patient satisfaction. In this context, a study showed that automated drug-dispensing systems carry advantages over traditional systems in improving inventory control and reducing storage errors, resulting in better patient satisfaction [37,38].

The participants in this study were satisfied with the perceived accuracy of the pharmacy’s handling of medical prescription information. In support of the claim that prescription accuracy contributes to patient satisfaction, a systematic review study revealed that the integration of e-prescription and e-dispensing systems significantly reduced pharmacy errors [39]. This indicates that the adoption of automated systems may enhance perceived accuracy and is associated with higher levels of patient satisfaction and perceived safety.

### Patients’ Satisfaction With Medication Counseling and the Dispensing Process

Despite the growing use of e-prescription and e-dispensing systems, challenges remain in their utilization. The findings of this study indicated that the patients reported high levels of satisfaction with medication counseling overall. Specifically, pharmacists were commended for clearly explaining the purpose of medication use, providing accurate dosage instructions, and allocating sufficient time for patients during consultations. However, approximately half of the participants expressed dissatisfaction with the counseling received regarding potential drug side effects and proper medication storage.

A US study into outpatient use of electronic prescriptions found that the use of an e-prescribing system led to lower rates of adverse drug events among patients, suggesting that appropriate counseling on side effects and storage could further reduce such risks [40]. Therefore, in this study, the lack of counseling regarding the side effects of medications and storage conditions indicates a potential gap in quality of practice. This may pertain to numerous reasons, such as a long queue of patients waiting, leading to time pressures, or pharmacists’ limited counseling skills, which would affect the quality of the counseling. This shortcoming may reflect the need for additional pharmacist training to improve their counseling skills and not neglect any important information from patient education. Previous research has shown that training physicians in e-prescribing significantly reduces errors and improves workflow, thus indicating that e-prescription systems alone do not resolve counseling-related challenges unless supplemented by targeted education [41]. Addressing these aspects could significantly enhance both patient satisfaction and medication safety.

In addition, despite the automation of the dispensing process, the findings of this study reported longer waiting times for patients with prescribed multiple medications. This association suggests that prolonged waiting times may negatively influence patients’ overall perception of outpatient pharmacy domains, including pharmacists’ attitudes, medication counseling, medication supply, and dispensing medications. The literature showed that previous studies are consistent with the findings of this study, indicating that waiting time is a critical determinant of patient satisfaction, irrespective of whether outpatient pharmacy services are delivered through conventional or automated systems [38,42,43]. Therefore, the findings revealed that the success of automated outpatient pharmacy services relies not only on technology use but also on efficient workflow that led to suitable time medication dispensing.

This finding highlights the need for process optimization within pharmacy workflows. As with counseling gaps, delays in dispensing may diminish the perceived benefits of automation. Similar studies conducted in Saudi Arabia [38,44] emphasized the value of integrated pharmacy systems while acknowledging the need for region-specific improvements. These studies support the idea that continuous pharmacist education and the implementation of structured feedback mechanisms are essential for identifying and addressing system inefficiencies. By incorporating such strategies, pharmacies can better meet patient expectations and improve the outpatient pharmacy services.

### Impact of E-Prescribing and E-Dispensing Systems on Outpatient Pharmacy Services

This study found that most patients reported a positive impact based on their experiences with the medication dispensing process, with pharmacist communication emerging as a key contributor to patient satisfaction. These findings are consistent with those of a study in Qatar in 2013, which emphasized the importance of pharmacist-patient communication [16]. While pharmacy staff provide irreplaceable expertise in prescription monitoring and dispensing, the integration of electronic systems may help alleviate workload and reduce cognitive burden. Participants perceived that reducing manual tasks enhanced efficiency and contributed to improved patient safety by minimizing the risk of human error [45]. Pharmacists and pharmacy technicians are susceptible to fatigue, stress, and other human limitations, which may compromise dispensing accuracy. In this context, e-prescription systems serve as a valuable support tool that may help mitigate such risks [18,45]. By providing an additional layer of safety and reliability, these systems can contribute to patients’ perceptions of positive outcomes and higher satisfaction levels.

The absence of a comparison group involving nonautomated outpatient pharmacies is considered a key methodological limitation of this study. Consequently, the findings cannot isolate the specific effects of automation from other contextual or organizational factors and should be interpreted as reflecting patient experiences within automated pharmacy settings only, which limits their generalizability to other pharmacy models. Future studies using comparative or quasi-experimental designs are needed to evaluate the incremental impact of automation on patient experience and service performance.

### Limitations

This study has several limitations. First, it focused exclusively on patients in Kuwait. While the findings provide valuable insights into local users of outpatient pharmacy services, the generalizability of the results to other populations and health care systems may be limited. In addition, the absence of a comparison group involving nonautomated outpatient pharmacies limits the ability to isolate the specific impact of automation.

Second, patient satisfaction data were self-reported, which may have introduced recall or self-reporting bias and may not accurately reflect the actual quality or safety of outpatient pharmacy services. Third, the presence of pharmacy students in the waiting area may have introduced courtesy or social desirability bias, which may have potentially inflated satisfaction scores (approximately 95% agreement), despite private survey completion. The consistently high levels of agreement observed may therefore partly reflect response tendencies rather than true satisfaction levels. Accordingly, future studies should consider alternative data collection approaches or comparative analyses across sites or time periods to better assess this potential bias. Fourth, despite the sensitivity analyses conducted, voluntary participation and convenience sampling may have introduced selection bias, as patients who were more satisfied or more engaged with automated outpatient pharmacy services may have been more likely to participate. Although the sensitivity analyses suggest that potential underrepresentation of less satisfied patients is unlikely to have materially altered the study conclusions, the findings may not be fully representative of all users of automated outpatient pharmacy systems in Kuwait, particularly individuals who actively avoid or disengage from these systems. Fifth, the study was conducted in 4 governmental hospitals and did not include private hospitals or pharmacies, which may differ in service quality, system implementation, and operational policies. Sixth, the cross-sectional design captured perceptions at a single point in time, limiting the ability to assess changes in patient-reported satisfaction over time.

Finally, although responses were obtained from both patients and caregivers, caregivers represented a smaller proportion of the sample (104/416, 25%). An exploratory comparison using the Mann-Whitney *U* test did not detect statistically significant differences between the 2 groups; therefore, further subgroup analyses were not pursued.

### Future Research

Future research should link patient-reported perceptions with objective safety and quality indicators, such as medication dispensing error rates, adverse drug events, dispensing accuracy, workflow measures, and prescription turnaround time, to determine whether perceived benefits correspond to measurable improvements in patient outcomes. Longitudinal and mixed methods designs, including qualitative interviews, may provide deeper insight into how and why perceived benefits evolve over time and whether anticipated benefits are realized in routine practice. Methodologically, probability-based sampling approaches should be considered to improve representativeness and reduce selection bias. In addition, remote or independent survey administration and comparative designs across data collection settings may help assess and minimize social desirability bias. Comparative studies involving governmental and private sector hospitals or pharmacies are also warranted, given potential differences in implementation maturity, staffing, workflows, and service policies. Finally, future studies specifically powered to compare patient and caregiver perspectives, alongside technical and organizational assessments, would provide a more comprehensive evaluation of system effectiveness and help identify actionable improvement strategies.

### Conclusions

This study demonstrated that integrated e-prescribing and e-dispensing systems offer clear perceived benefits in outpatient pharmacies within governmental hospitals in Kuwait. This is reflected in the positive experiences reported by patients, reflecting overall perceived satisfaction and perceived service performance within the operating systems.

However, the findings also highlight areas requiring improvement to maximize patients’ satisfaction toward pharmacy automation systems. The following clinical implications are made:

1. Workflow design optimization: operational strategies should be implemented to improve pharmacy workflow design within automated pharmacy settings to enhance work efficiency, especially for processing lengthy prescriptions and managing drug inventories to reduce dispensing delays, as well as making sure that a counseling area is available for patient education.
2. E-prescribing system enhancement: counseling prompts should be integrated to ensure that important information is delivered completely and properly to patients.
3. Automated inventory management: enhanced inventory control should be implemented, including proactive stock monitoring to clarify what is available and what is near expiry to improve the supply chain management. This is essential because the results showed that approximately 25% (105/416) of patients reported medication unavailability.
4. Staff training: pharmacy staff should receive comprehensive training on best practices for using integrated e-prescription and e-dispensing systems. This would improve patient counseling, interprofessional communication, patient safety, and overall patient satisfaction.
5. Feedback mechanisms: regular collection of feedback from both patients and pharmacy staff should be instituted to identify and address issues related to waiting times, medication availability, and the quality of counseling services.

By addressing these identified gaps, pharmacists can enhance patient satisfaction and improve the overall performance of outpatient pharmacy services supported by integrated digital systems. Strengthening the digital infrastructure and workflow integration would enable hospitals to optimize outpatient pharmacy services and move toward patient-centered digital pharmacy models that support efficient, reliable, and safe medication services. These findings have relevance beyond the study setting and may inform the development of digital outpatient pharmacy services across hospitals in the Arabian Gulf region.

## Supplementary material

10.2196/80963 Multimedia Appendix 1 The impact of integrated e-prescribing and e-dispensing systems on outpatient pharmacy services.

10.2196/80963 Multimedia Appendix 2 Overall satisfaction with automated outpatient pharmacy services.

## Abbreviations

e-dispensing: electronic dispensing
e-prescribing: electronic prescribing
HIS: hospital information system
KMO: Kaiser-Meyer-Olkin
