# Supplementary figures and images for: Patient Experiences With Outpatient Pharmacy Services in Hospitals Using Automated Pharmacy Systems: Cross-Sectional Study
Source: JMIR Med Inform. 2026 Mar 6;14:e80963. doi: 10.2196/80963 (PMC12978900; doi:10.2196/80963)

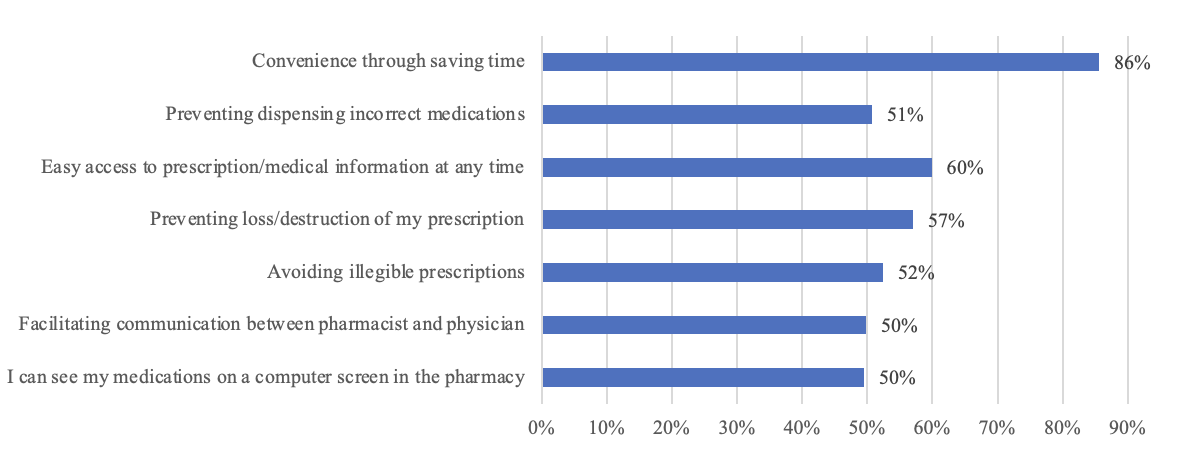

Supplement: Multimedia Appendix 1 [file medinform-v14-e80963-s001.png]

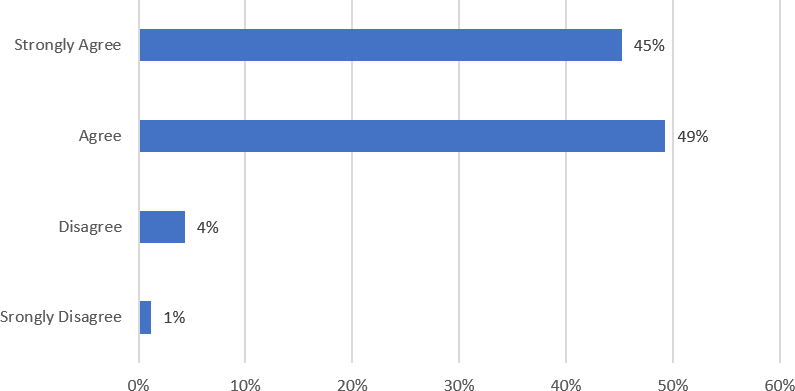

Supplement: Multimedia Appendix 2 [file medinform-v14-e80963-s002.png]
